# Atypical Case of Minoxidil-Induced Generalized Anasarca and Pleuropericardial Effusion

**DOI:** 10.7759/cureus.15424

**Published:** 2021-06-03

**Authors:** Wahab A Gbadamosi, Jacqueline Melvin, Marvin Lopez

**Affiliations:** 1 Radiology, Coliseum Medical Centers, Macon, USA; 2 Internal Medicine, Coliseum Medical Centers, Macon, USA

**Keywords:** medication side effect, minoxidil, pleuropericardial effusion, generalized anasarca, painful swelling, exertional dyspnea

## Abstract

Minoxidil is an antihypertensive medication used to control blood pressure that is resistant to three or more antihypertensive medications including diuretics. There have only been a few reported cases of minoxidil-induced pleuropericardial effusion with anasarca. Here, we discuss the case of a 70-year-old male with a history of uncontrolled hypertension who presented to the hospital with swelling of the extremities. He was on minoxidil 10 mg twice a day and complained of generalized body swelling and extremity pain with symptoms of dyspnea on exertion, paroxysmal nocturnal dyspnea, weight gain, abdominal distention, and intermittent, throbbing extremity pain (8/10). The patient denied a history of cardiac disease, pulmonary embolism, deep vein thrombosis, and malignancy. His vitals were stable with a blood pressure of 152/67 mmHg. The physical examination findings were significant for positive jugular vein pressure, bilateral crackles at the lung bases with abdominal distention, fluid thrill, and flank edema. The extremities showed pitting edema in the upper and lower extremities that were tender to palpation. Laboratory results were only significant for uremia and elevated brain natriuretic peptide. Electrocardiography, echocardiography, and CT of the chest and abdomen exhibited findings consistent with pericardial effusion, pleural effusion, abdominal ascites, and anasarca in the soft tissues of the axilla and chest wall. After discontinuation of minoxidil and starting intravenous diuretics, the patient showed clinical improvement. This case report reviews and explains that minoxidil-induced anasarca should be considered as a differential diagnosis in patients taking minoxidil as knowledge of this rare finding may lead to early diagnosis and management.

## Introduction

Resistant hypertension (RHTN) is a severe medical condition that is estimated to occur in 9-18% of patients with hypertension [1,2]. It is characterized by its lack of response to treatment with three or more antihypertensive medications, including a diuretic. Minoxidil was approved in the 1970s as one of the medications indicated for the management of RHTN. However, it carries a boxed warning of a rare finding: medication-induced pericardial effusion that can progress to tamponade [3]. There have been case reports of minoxidil used in dialysis and normal renal patients and causing pericardial effusion, but only three cases of pleuropericardial effusion have been published and reported to date [4-6]. This case report discusses minoxidil-induced anasarca and pleuropericardial effusion. Knowledge of this rare clinical association may lead to early diagnosis and management.

## Case presentation

A 70-year-old male with a history of uncontrolled hypertension presented to the hospital. He was on minoxidil 10 mg twice a day and complained of generalized body swelling and bilateral extremity pain. He had been in his usual state of health until approximately a week before hospital presentation when he noticed swelling in both lower extremities that progressively spread to both upper extremities. There was associated dyspnea on exertion, paroxysmal nocturnal dyspnea, weight gain, and abdominal distention. The patient also reported intermittent, throbbing pain (8/10) exacerbated by ambulation and movement of the upper extremities. He denied any history of cardiac disease, pulmonary embolism, deep vein thrombosis, or malignancy. He further denied fever, cough, hemoptysis, night sweats, dizziness, or palpitations.

The patient’s medical history was significant for hypertension, gastroesophageal reflux disease, appendectomy, and cholecystectomy. He had no known drug allergies and his medications included minoxidil for three years, clonidine, and pantoprazole. He reported a 30-pack-year smoking history with occasional alcohol use but denied recreational drug use. Family history was significant for cardiovascular disease.

On examination, the patient’s temperature was 36.9°C, heart rate was 80 beats per minute, blood pressure was 152/67 mmHg, respiratory rate was 18 beats per minute, and oxygen saturation was 99% on room air. He appeared in mild distress. He was alert and oriented to self, time, place, and situation. The patient’s pupils were round and reactive to light. He was anicteric with moist mucous membranes, a supple neck, and a positive jugular vein pressure. The lung examination was significant for bibasilar crackles. His heart had a regular rate and rhythm, as well as normal S1 and S2 sounds, with 1/6 systolic murmur present at the mitral area. The abdomen was soft with normal bowel sound but distended with positive fluid thrill and flank edema. There was no guarding or costovertebral region tenderness. The extremities showed pitting edema in the upper and lower extremities and were tender to palpation. The neurology examination and the results of the remaining examinations were unremarkable.

The comprehensive metabolic panel was unremarkable except for elevated blood urea nitrogen 28 (6-18) and serum creatinine 1.9 (0.7-1.3). The complete blood count was significant for leukopenia 3.2 K/uL (4-11 K/uL), hemoglobin of 7.9 g/dL, hematocrit of 24.95, mean corpuscular volume of 89.1, and platelet of 167 K/uL (140-440 K/uL). Brain natriuretic peptide was elevated at 290 mg/dL (0-100 mg/dL). His HbA1c was also elevated at 7.5%. Troponin level was normal at 18 pg/mL (0-53 pg/mL). Additionally, urinalysis was unremarkable; hepatic serology was nonreactive for hepatitis; albumin was 3.5 g/dL (3.5-5.0 g/dL); and total protein was within normal limits at 5.9 g/dL (5.7-8.2 g/dL). Further testing revealed normal thyroid function. Malignancy workup for B19, prostate-specific antigen, carcinoembryonic antigen, alpha-fetoprotein, and cancer antigen 19-9 as well as infectious etiology were all negative.

**Figure 1:**
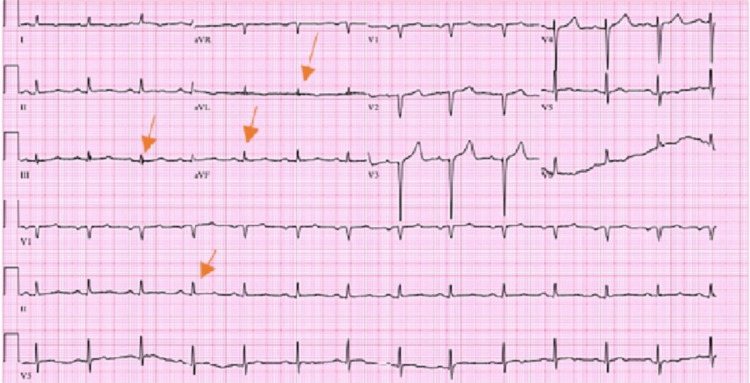
Electrocardiogram with low-voltage QRS on multiple leads.

**Figure 2:**
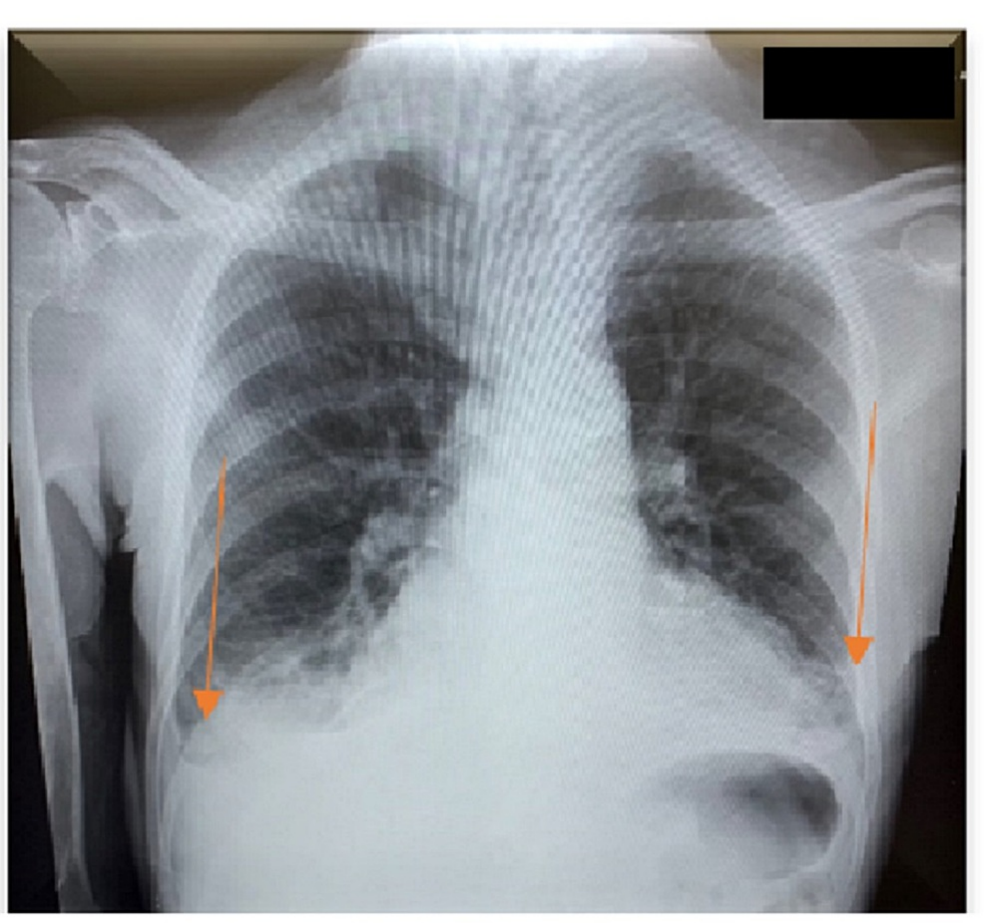
Chest X-ray with bilateral basilar pleural opacities.

**Figure 3:**
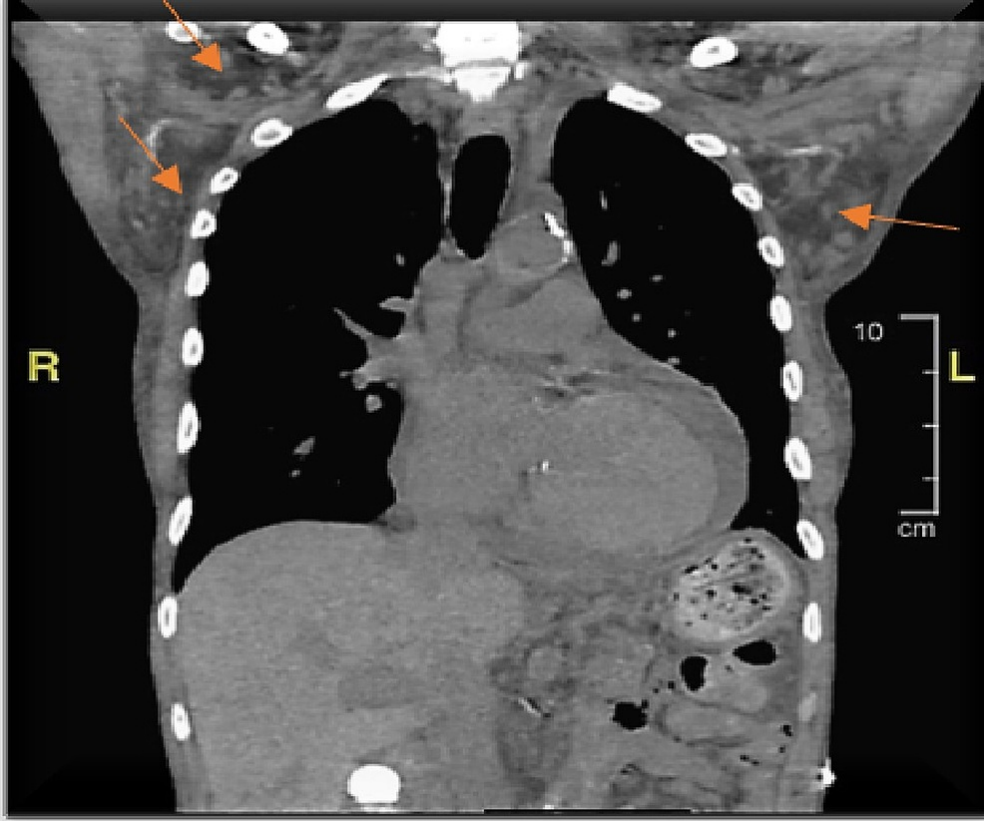
CT chest with fluid in the soft tissue of the axilla and chest wall.

**Figure 4:**
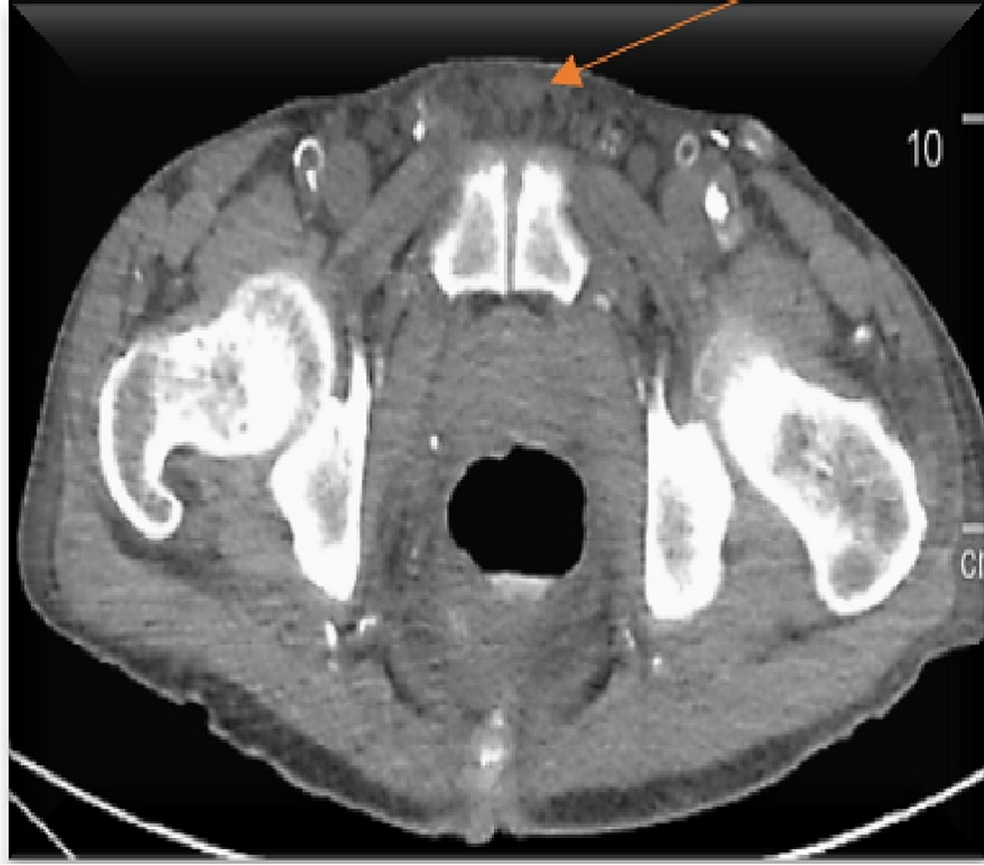
CT pelvic with fluid in the soft tissues.

**Figure 5:**
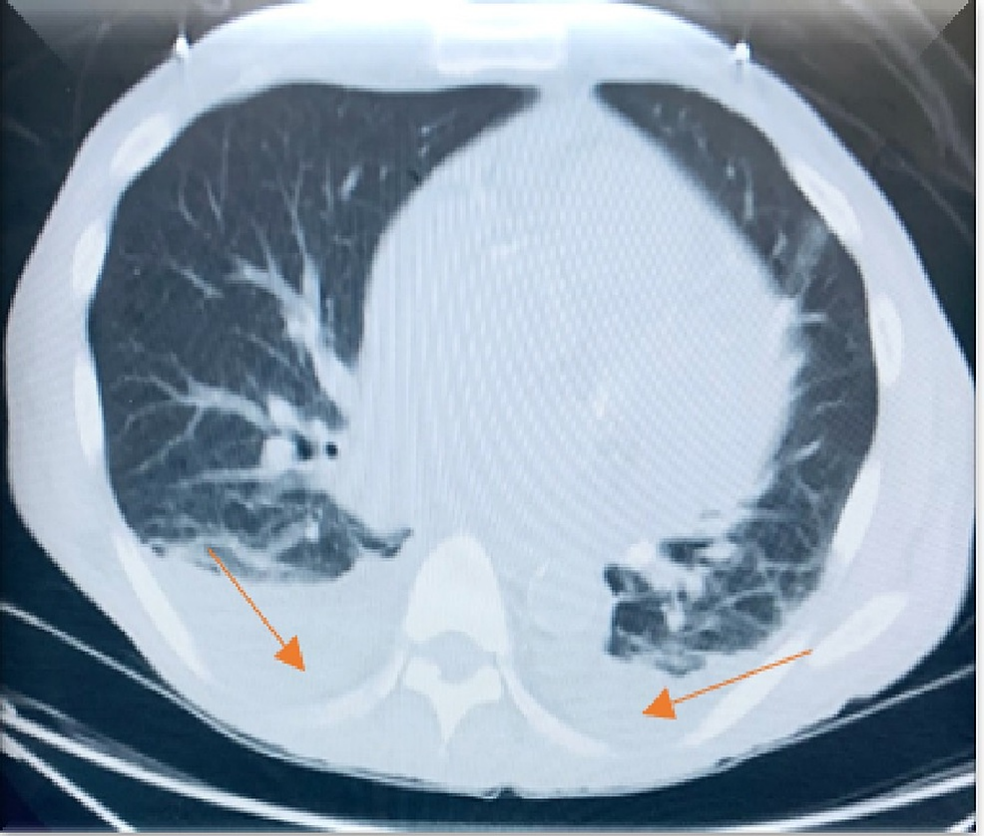
CT chest lung window showing bilateral pleural effusion.

**Figure 6:**
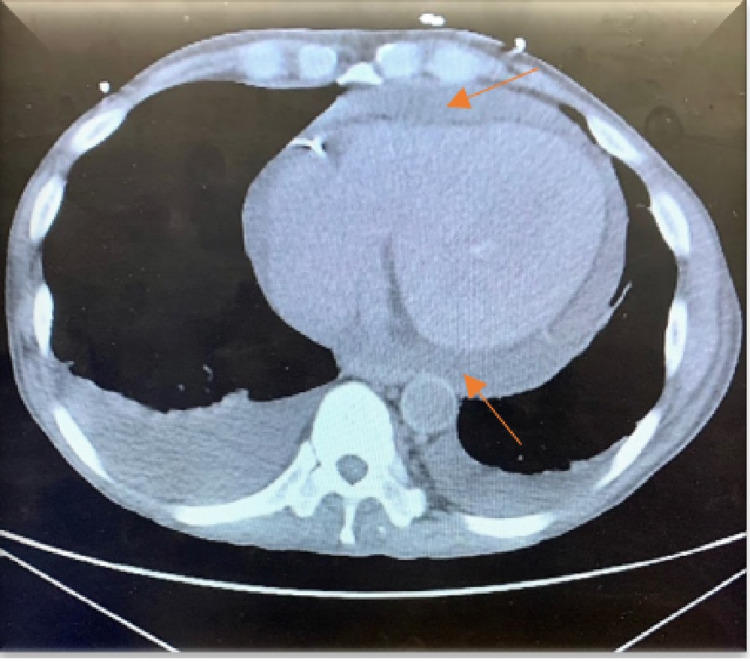
CT chest soft tissue window showing pericardial effusion.

**Figure 7:**
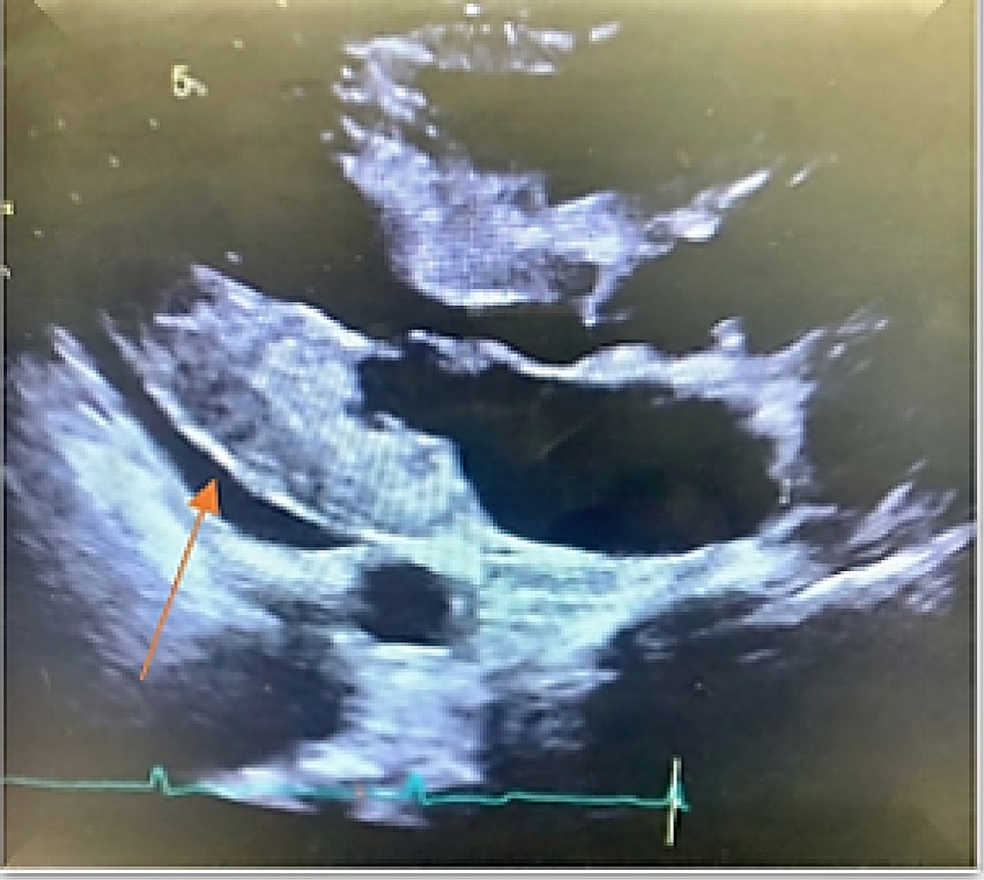
Two-dimensional echocardiography long-axis view showing pericardial effusion.

**Figure 8:**
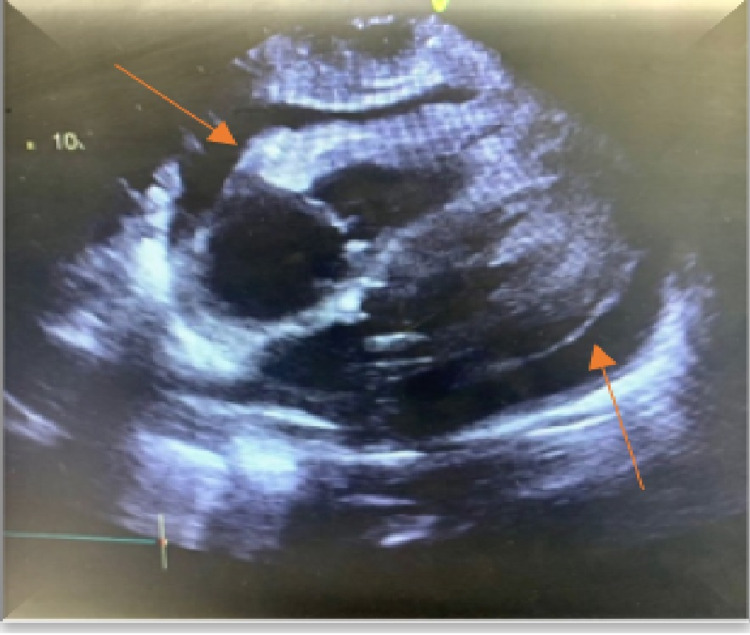
Two-dimensional echocardiography apical four-chamber view showing pericardial effusion.

An electrocardiogram showed normal sinus rhythm, no ST/T wave changes, and low-voltage QRS (Figure 1). Doppler ultrasound was negative for thrombosis in either extremity. The chest radiograph showed bilateral basilar pleural opacities (Figure 2). CT of the chest showed a large pleuropericardial effusion and anasarca in the soft tissues of the axilla and chest wall (Figures 3, 5, 6). Computed tomography of the abdomen showed ascites in the pelvis and edema of the subcutaneous tissue (Figure 4). Two-dimensional transthoracic echocardiography showed moderate free-flowing pericardial effusion with features not indicative of tamponade (Figures 7, 8). The estimated ejection fraction was 60% with no wall abnormality, normal valve functions, and pulmonary systolic pressure.

Throughout the hospital course, specialists in cardiology, nephrology, gastroenterology, and oncology were consulted. Concerns for malignancy, liver disease, cardiovascular, and pulmonary etiology were low after extensive workup. The patient’s anasarca and pleuropericardial effusion were attributed to minoxidil. The team discontinued minoxidil and started an intravenous dose of furosemide 80 mg, oral metolazone 5 mg daily, and fluid restriction. The patient showed clinical improvement in dyspnea, generalized anasarca, and extremity swelling. The patient’s clinical symptoms improved over the hospital course, and he was discharged home with an outpatient diuretic, beta-blocker, and a schedule of follow-up visits. At his four-week follow-up, a repeat two-dimensional echocardiogram showed complete resolution of pericardial effusion and his symptoms.

## Discussion

The differential diagnoses of pleuropericardial effusion and generalized anasarca include cardiac disease, hepatic disease, protein-calorie malnutrition, thyroid disease, obstructive sleep apnea, renal etiology, medications such as calcium channel blockers, corticosteroids, vasodilators, and malignant ascites [1,7]. For a patient with generalized anasarca, most diagnoses are made based on history and physical findings. However, if this is not consistent with a particular etiology, we tend to obtain further evaluation and workup. For the patient described in this report, we performed an extensive workup, including cardiac, pulmonary, hepatic, renal, gastrointestinal, and malignancy workup, but none were successful in assisting us in finding a diagnostic result.

Minoxidil is an oral direct vasodilator antihypertensive agent that was approved in the 1970s for the treatment of RHTN [1,8]. RHTN is a medical condition in which a patient fails therapy management to three or more antihypertensive drugs at maximum dosage after exclusion of pseudohypertension [2]. Minoxidil is a prodrug that is metabolized into minoxidil sulfate, which then opens the adenosine triphosphate-sensitive potassium channel in the vascular smooth muscle of the arteriolar, leading to vasodilation with little to no effect on veins [3,8]. About 90% of minoxidil is absorbed from the gastrointestinal tract and metabolized by hepatic glucuronidation at the N-oxide position of the pyrimidine ring to its active metabolite [8]. The average plasma half-life is around three to four hours, and it is excreted via the kidney [3]. When minoxidil is used for the treatment of RHTN, it is recommended to be given together with therapeutic doses of beta-blockers to prevent tachycardia and increase myocardial oxygen demand along with a diuretic, which acts at the ascending loop of Henle, to prevent fluid accumulation [3]. Our patient in this case had been diagnosed with RHTN, prescribed minoxidil, but without a beta-blocking and diuretic agent, and was lost to follow-up with the medical provider. This could explain his pleuropericardial effusion and anasarca given that it is recommended that a therapeutic dose of beta-blockers along with a loop diuretic be given to decrease salt and water retention [1].

One of the adverse effects of minoxidil is pericardial effusion that can progress into tamponade via fluid retention [3]. The mechanism of fluid retention is a combination of dose-dependent, neurohormonal changes and renal hemodynamic [8]. The recommended starting dose is 5 mg per dose, and the maximum dose is 100 mg per day [1,3]. It can be titrated every three days by doubling the daily dose up to the maintenance range of 10-40 mg per day [1,3,8]. When used above the recommended dose, it can lead to an increased adverse effect. Furthermore, minoxidil can also lead to the activation of the renin-angiotensin axis, which prompts increased biosynthesis of aldosterone and leads to increased plasma and urinary aldosterone, resulting in fluid retention [9,10]. Lastly, renal hemodynamic fluid retention from the activation of the potassium channel in the ascending limb of the nephron increases the Na^+^/2Cl^−^/K^+^ co‐transporter activity and, thereby, increases sodium and chloride reabsorption [11]. In our patient, who was on a maintenance dose of 20 mg per day, the exact mechanism of pericardial, pleural effusions, and anasarca is not clearly understood but might be attributed to any of these mechanisms.

The management of anasarca and pleuropericardial effusion involves the management of hemodynamic stability and the treatment of primary etiology. Pleuro-pericardiocentesis is required in a hemodynamically unstable patient [1]. Patients who are hemodynamically stable with no evidence of tamponade do not require immediate drainage of effusion [12]. In the case of the patient discussed here, minoxidil was discontinued, both intravenous and oral doses of a diuretic agent, along with beta-blocker, and fluid restriction was initiated.

## Conclusions

Minoxidil is an oral antihypertensive agent that is indicated for RHTN. It works primarily on the arteriole smooth muscle and can cause salt and water retention leading to fluid accumulation in the soft tissue and internal organs, especially around the heart and lung. Both the Food and Drug Administration and the drug manufacturer warn against this risk, recommending the addition of a beta-blocker and diuretic. Therefore, in conjunction with what was learned from this case study, it is highly recommended that this protocol be followed under the close supervision of a physician.
